# Suppression of *him-14(it44ts)* by a transgene insertion expressing GFP::COSA-1

**DOI:** 10.17912/micropub.biology.000430

**Published:** 2021-08-24

**Authors:** Chloé Girard, Chantal C Akerib, Anne M Villeneuve

**Affiliations:** 1 Departments of Developmental Biology and Genetics, Stanford University School of Medicine, Stanford, CA 94305, U.S.A.; 2 current affiliation: Université Paris-Saclay, CEA, CNRS, Institute for Integrative Biology of the Cell (I2BC), 91198, Gif-sur-Yvette, France

## Abstract

Meiotic crossover formation requires the activity of multiple pro-crossover factors, including the MutSγ complex and the cyclin-related protein COSA-1, that become concentrated together at the sites of crossover recombination intermediates. Here we show that a transgene insertion expressing GFP::COSA-1 can suppress the crossover deficit caused by a partial reduction in MutSγ function. Our data, combined with previous findings, support a model in which COSA-1 promotes crossover formation, at least in part, through positive regulation of MutSγ function.

**Figure 1. Suppression of the him-14(it44ts) crossover deficit at semi-permissive temperature by the GFP::COSA-1-expressing transgene insertion meIs8:**
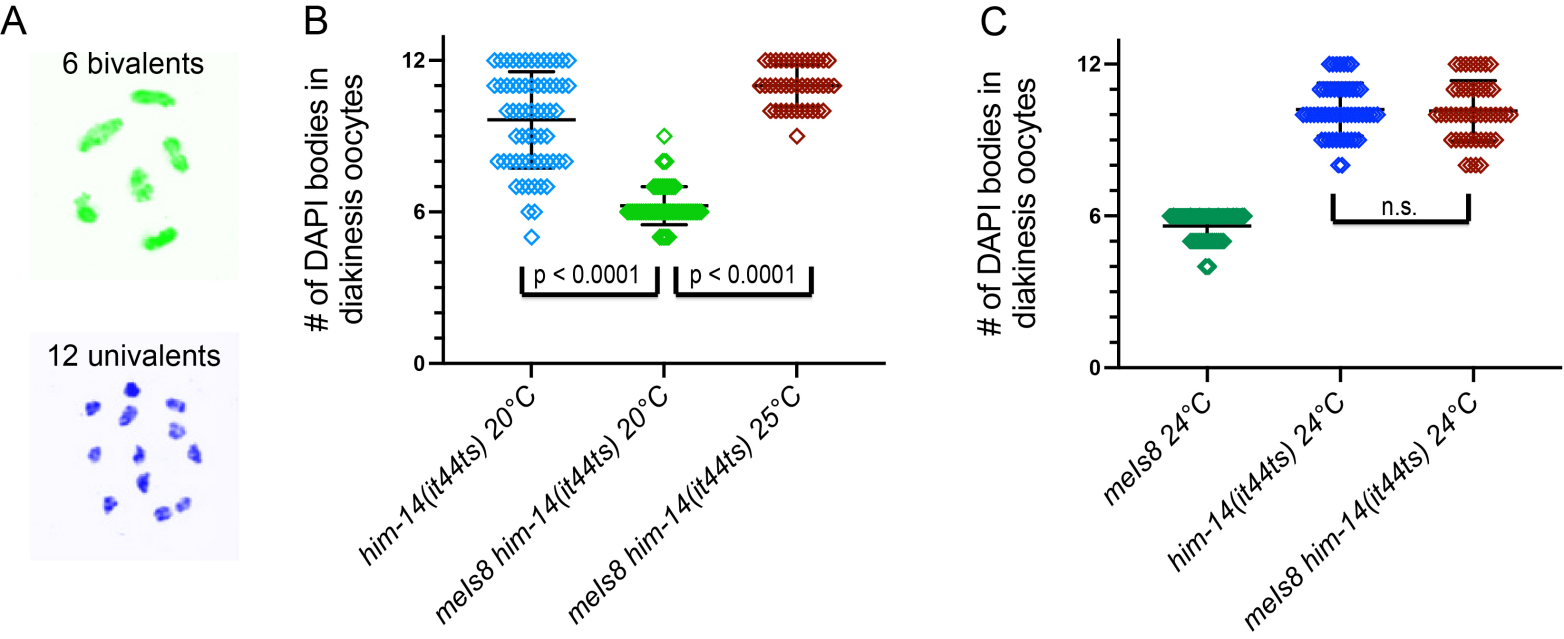
A) Example oocyte karyotypes (modified from Zalevsky *et al.* 1999) illustrating a normal wild-type karyotype (top) with all 6 pairs of homologous chromosomes connected by chiasmata (6 bivalents), and a karyotype from a recombination-deficient mutant (bottom) with 12 achiasmate chromosomes (12 univalents). B and C) Graphs quantifying numbers of resolvable DAPI-stained bodies in diakinesis oocyte nuclei; each diamond represents a nucleus, and mean and standard deviation are indicated. *him-14(it44ts)* is a temperature-sensitive mutation affecting the HIM-14/MSH-4 subunit of the MutSγ complex; *meIs8* is a transgene insertion expressing a GFP::COSA-1 fusion protein. Fixation and DAPI staining were conducted as in Bessler *et al.* 2007, and numbers of DAPI bodies were counted in oocyte nuclei in the -1 to -3 positions. For the 20°C data, worms raised at the semi-permissive temperature of 20°C were fixed at 24 h post L4; for the 24° and 25°C data, worms were raised at 20°C until the L4 stage, then were shifted to 24° or 25°C for 24 h before fixation. (Note that this assay tends to underrepresent the actual numbers of DAPI bodies present, as some chromosomes may be too close together to be resolved unambiguously.) Genotypes and numbers of nuclei scored: B) *him-14(it44ts) unc-4* 20°C*,* n = 62; *meIs8 him-14(it44ts) unc-4* 20°C, n = 65; *meIs8 him-14(it44ts) unc-4* 25°C, n = 40; C) *meIs8* 24C°, n = 63; *him-14(it44ts)* 24°C*,* n = 53; *meIs8 him-14(it44ts)* 24C°, n = 47. p-values are for two-sided Mann Whitney tests. Because of the temperature-sensitive nature of *him-14(it44ts)*, all plotted data for each graph are derived from cohorts of worms raised, fixed and stained in parallel in the same experiment. The finding that *meIs8* suppresses the *him-14(it44ts)* CO deficit at 20°C has been replicated in at least four independent experiments. The findings that *meIs8 him-14(it44ts) unc-4* is severely defective in CO formation at 25°C and that suppression of *him-14(it44ts)* by *meIs8* is abrogated at 24C° have each been replicated in at least two independent experiments.

## Description

Formation of crossovers (COs) during meiosis depends on the coordinated activities of multiple pro-CO factors, including the MutSγ complex and COSA-1, that become concentrated at the sites of CO-designated recombination intermediates in an interdependent manner (Yokoo *et al.* 2012; Woglar and Villeneuve 2018). The MutSγ complex is a heterodimer comprising the conserved proteins MSH-5 and MSH-4 (aka HIM-14, encoded by the *him-14* gene) (Zalevsky *et al.* 1999; Kelly *et al.* 2000). COSA-1 is a member of the cyclin superfamily and is hypothesized to function as part of a cyclin-dependent protein kinase (CDK) complex (Yokoo *et al.* 2012). The MutSγ complex was suggested to be a potential target for this hypothesized COSA-1 dependent CDK activity, as MSH-5 has multiple potential CDK phosphorylation sites and can be phosphorylated *in vitro* by a mammalian CDK. These and other data led to a model in which COSA-1-dependent phosphorylation boosts the activity of MutSγ as part of a positive feedback loop that concentrates pro-CO factors at CO sites (Yokoo *et al.* 2012).

In the course of constructing strains for experiments using the temperature-sensitive missense mutation *him-14(it44ts)*, we obtained additional evidence consistent with a role for COSA-1 as a positive regulator of MutSγ function. Specifically, we found that the presence of *meIs8,* a transgene insertion expressing a functional GFP::COSA-1 fusion protein (Yokoo *et al.* 2012), resulted in significant rescue of the CO deficit caused by the *him-14(it44ts)* mutation at 20°C, a semi-permissive temperature at which MutSγ activity is reduced but not eliminated. We evaluated the success or failure of CO formation by examining DAPI-stained bodies present in oocytes at diakinesis, the last stage of meiotic prophase. Wild-type oocytes contain six DAPI bodies, reflecting six connected homolog pairs (bivalents) harboring CO-based connections (chiasmata), whereas up to 12 DAPI bodies can be resolved in mutants lacking COs, reflecting unconnected individual chromosomes (univalents). In contrast to the high frequency of univalents observed in *him-14(it44ts)* mutant oocytes at the semi-permissive temperature of 20°C, six bivalents (indicative of successful CO formation for all homolog pairs) were observed in most *meIs8 him-14(it44ts)* oocytes at 20°C. This finding suggests that an elevated level of COSA-1 protein, due to the presence of the transgene insertion expressing GFP::COSA-1, can increase the likelihood of CO formation in the context of reduced MutSγ activity. However, suppression of the *him-14(it44ts)* CO defect does not occur at the more restrictive temperatures of 24°C or 25°C where activity of MutSγ is severely impaired. We note that six DAPI bodies are consistently observed at 24°C in diakinesis oocytes of AV818 worms in which *meIs8* is the only source of COSA-1 activity (mean = 6.0 ± 0.0, n = 72), indicating that functional GFP::COSA-1 is expressed from *meIs8* at 24°C. Together our data suggest that the presence of *meIs8* does not bypass the requirement for MutSγ activity to form COs, but instead suppresses *him-14(it44ts)* by augmenting the residual MutSγ activity present at semi-permissive temperature . Our data are consistent with a model in which COSA-1 promotes CO formation, at least in part, through positive regulation of MutSγ.

## Reagents

**Table:** 

**Strain**	**Genotype**	**Available from**
KK323	*him-14(it44ts) unc-4(e120)/mnC1 II*	AV lab
AV893	*meIs8[unc-119(+) pie-1promoter::gfp::cosa-1] him-14(it44ts) unc-4(e120) II*	AV lab
AV630	*meIs8[unc-119(+) pie-1promoter::gfp::cosa-1] II*	CGC
AV902	*him-14(it44ts) II*	AV lab
AV956	*meIs8[unc-119(+) pie-1promoter::gfp::cosa-1] him-14(it44ts) II*	AV lab
AV818	*meIs8[unc-119(+) pie-1promoter::gfp::cosa-1] cosa-1(tm3298) II*	AV lab
